# Awareness and Knowledge of the General Population About Monkeypox Disease in Riyadh, Saudi Arabia

**DOI:** 10.7759/cureus.50171

**Published:** 2023-12-08

**Authors:** Nora M Alhummayani, Jarah M Alobaid, Ibrahim M Altamimi, Turki A Nuwayim, Kholood K Alyanbaawi, Nouf M Alhomayani, Hatim M Alhamyani

**Affiliations:** 1 Pediatrics, King Saud Medical City, Riyadh, SAU; 2 General Practice, Ministry of Health, Riyadh, SAU; 3 General Practice, First Health Cluster, Ministry of Health, Riyadh, SAU; 4 Family Medicine, Ministry of Health, Riyadh, SAU; 5 Pharmacy, Taif University, Taif, SAU

**Keywords:** awareness, virus transmission, general population, knowledge, monkeypox

## Abstract

Background

Monkeypox is a globally spreading disease, representing a significant threat to human life in all countries of the world, and Saudi Arabia is no exception. This study aimed to assess the knowledge of the general adult population in Riyadh, Saudi Arabia about monkeypox.

Methodology

This cross-sectional study was conducted in Riyadh, Saudi Arabia among a sample of adults (aged over 18 years) who attended the outpatient clinics of the First Cluster primary healthcare centers, King Saud Medical City, and Ministry of Health in November 2022. A self-administered Arabic questionnaire, including sociodemographic characteristics of the participants, beliefs regarding monkeypox, and knowledge assessment about monkeypox through 23 multiple-choice questions, was utilized.

Results

The study included 375 participants, of whom 270 (72%) were aged between 26 and 45 years, and 195 (52%) were males. Overall, 258 (68.8%) participants had insufficient knowledge about monkeypox. Multivariate logistic regression analysis revealed that participants aged over 45 years were almost at a three-fold risk of having an insufficient level of knowledge about the disease compared to those aged between 18 and 25 years (adjusted odds ratio (AOR) = 3.03, 95% confidence interval (CI) = 1.07-8.69, p = 0.037). University/higher educated participants were at 61% lower risk of having an insufficient level of knowledge about the disease compared to those with an education level of below secondary school (AOR = 0.39, 95% CI = 0.09-0.65, p = 0.013). Compared to housewives/non-working participants, governmental employees were at a significantly lower risk of having insufficient knowledge about monkeypox (AOR = 0.48, 95% CI = 0.26-0.89, p = 0.020).

Conclusions

Knowledge of the general population in Riyadh, Saudi Arabia regarding monkeypox is insufficient, particularly regarding the fact that a monkeypox vaccine is available in Saudi Arabia and monkeypox is not a new infection that appeared in 2022. Therefore, there is a need to implement educational sessions at primary care centers and outpatient clinics of hospitals about the disease.

## Introduction

Monkeypox is a rare zoonotic disease caused by the monkeypox disease virus (MPXV), a member of the genus Orthopoxvirus, which belongs to the same family of virus that causes smallpox [1]. Monkeypox is a non-fatal disease that presents with fever accompanied by malaise, fatigue, headache, back pain, and skin rash, which are similar to those of smallpox, except for lymphadenopathy [2,3]. Moreover, the disease is characterized by its vesiculopustular rash with all stages (macular, papular, vesicular, and pustular), which differentiate it from other diseases with vesiculopustular rash [2,3].

Monkeypox has an incubation period of 5-21 days. Its main transmission route is via droplets and contact with skin lesions or bodily materials and fluids contaminated with the virus [4,5]. However, maternofetal transmission has been reported [6,7]. Symptoms disappear spontaneously in two to three weeks [4,8].

Certain behaviors that require a high level of human-human contact pose a potential risk of transmitting the virus, including sleeping in the same room or bed, living in the same household, or drinking or eating from the same dish [5]. Surprisingly, no significant associations have been identified in laundering clothes, helping to clean the body, or helping in visiting the toilet. The theory behind the increased risk of certain behaviors could be that the virus can be more easily transmitted to the oral mucosa compared to activities that involve only exposed skin [5].

Human monkeypox disease was first discovered in the Democratic Republic of Congo in 1970 in a 12-year-old child. Although the disease was limited to West Africa, recently, new epidemics have been discovered in the United States, central Africa, and outside the African continent [6,9]. As most countries focussed on smallpox and its eradication, low attention to MPXV and its detection may have masked many infected cases. Since the World Health Assembly declared the global eradication of smallpox and cessation of routine smallpox vaccination programs, MPXV has been increasingly reported, especially among those who are not vaccinated with the smallpox vaccine as vaccination against Orthopoxvirus provides 85% protection against MPXV [6].

To date, there is no available vaccine or treatment for monkeypox as the Modified Vaccinia Ankara-Bavarian Nordic (MVA-BN) and tecovirimat drugs are still unavailable on a wide scale, despite being approved [10]. In addition, the cross-protection of childhood smallpox vaccines against monkeypox is limited among adults aged over 40 years. Moreover, younger individuals from non-endemic areas have lower immunity to monkeypox.

In Saudi Arabia, the first Monkeypox case was discovered in a person who came from Europe in July 2022 in Riyadh [11]. This study aims to assess the awareness and knowledge of the general adult population in Saudi Arabia about monkeypox and set recommendations to help educate the community on early preventive strategies.

## Materials and methods

Study design and setting

This cross-sectional study was conducted in Riyadh, which is the largest city and capital of Saudi Arabia, with a population of about 7.6 million people according to the 2019 census [12].

Study participants and eligibility criteria

Study participants were the general population who attended the outpatient clinics of the First Cluster primary healthcare centers (PHCCs), King Saud Medical City (KSMC), and the Ministry of Health (MOH) in Riyadh (N = 45 PHCCs). Participants included patients, caregivers, healthcare workers, administrators, and any person within the zone of PHCCs. Adults (aged over 18 years) of both genders and all nationalities were considered for inclusion in the study. Mentally disabled adults, those with severe physical problems, or those aged over 65 years were excluded. The period of data collection for the study (November 2022) constituted the target population.

Sample size estimation

The sample size was estimated with the assumption that the total population of adults attending outpatient clinics of the First Cluster PHCCs, KSMC in Riyadh in one month was more than 20,000 (as the sample size does not change much for populations larger than 20,000). The prevalence of good knowledge level regarding monkeypox was 48% in a recent study from Saudi Arabia [13]. Considering a confidence limit of 95% and a margin of error of 5%, the minimum required sample size was 377 patients. The sample size was computed using an online Roasoft sample size calculator [14].

Data collection tool and procedure

A two-stage sampling technique was adopted to select the required sample size. In the first stage, five PHCCs were selected using a simple random technique. In the second stage, the sample was equally distributed over the five selected PHCCs. Thus, almost 75 patients were selected from each PHCC through a systematic random sample (according to the total number of patients visiting each center daily) to select 10 patients from each center daily. Hence, about eight working days were needed for each center to select the required sample.

A self-administered Arabic questionnaire was utilized in this study. It consisted of three main parts, namely, sociodemographic characteristics of the patients (age, gender, residence, nationality, marital status, educational level, employment status, and monthly income in Saudi Riyals), beliefs regarding monkeypox (believing that monkeypox will affect social and economic life and thinking regarding the suggestion that monkeypox is a conspiracy or bioterrorism), and assessment of the knowledge about monkeypox through 23 multiple-choice questions with “Yes,” “No,” and “I don’t know” responses. It was validated in a previous Saudi study [13], and adopted from existing facts from the Centers for Disease Control and Prevention[15]. Correct responses were assigned a score of “1” whereas incorrect and don’t know responses were assigned a score of “0.” The total score and its percentage for each participant were computed. Patients who scored below 50% were considered to have “insufficient knowledge” whereas those who scored 50% or more were considered to have “sufficient knowledge” [16,17].

The researchers visited the involved PHCCs after obtaining approval from the MOH. The questionnaires were distributed to selected patients in these centers while they were waiting for their physician appointments after explaining the study goals. No names were disclosed to ensure confidentiality. Filled questionnaires were collected on the same day from each center. One or two centers were visited each day according to the circumstances.

Ethical considerations

Ethical approval for the study was obtained from the Institutional Review Board of KSMC (approval number: H1RE-04-Dec22-02). Before their involvement in the study, study aims were explained to all participants and informed consent was obtained verbally. Confidentiality was maintained throughout the study.

Data analysis

SPSS software version 28.0 (IBM Corp., Armonk, NY, USA) was used for data entry and analysis. Descriptive statistics (e.g., number, percentage, mean, range, standard deviation) and bivariate analytic statistics were computed using the chi-square test. Variables significant at the p <0.05 level were further analyzed by multiple regression analysis to determine the significant predictors of knowledge of monkeypox. P-values <0.05 were considered statistically significant.

## Results

The study included 375 participants out of the targeted 377 with a response rate of 99.5%. A total of 401 participants’ data were collected; however, due to incomplete questionnaires, 26 were excluded. Thus, a total of 375 questionnaires were included with a response rate of 99.5%. Overall, 270 (72%) participants were aged between 26 and 45 years. Slightly more than half of the participants (195, 52%) were males. The majority were Saudis (249, 93.1%) and living in urban areas (271, 98.9%). Overall, 230 (61.3%) participants were married, and 239 (63.8%) had a university/higher education. Governmental employees represented 160 (42.6%) participants while 103 (27.5%) were housewives/not working. In total, 153 (42.3%) participants had a monthly income ranging between 3,000 and 10,999 Saudi riyals (Table 1).

**Table 1:** Sociodemographic characteristics of the participants (N = 375). N: Number;%: percentage

Variables	N	%
Age in years		
18–25	52	13.9
26–45	270	72.0
>45	53	14.1
Sex		
Male	195	52.0
Female	180	48.0
Place of residence		
Urban area	371	98.9
Rural area	4	1.1
Nationality		
Saudi	349	93.1
Non-Saudi	26	6.9
Marital status		
Single	118	31.5
Married	230	61.3
Divorced/Widowed	27	7.2
Level of education		
Below high school	32	8.5
High school/Intermediate diploma	104	27.7
University/Higher education	239	63.8
Job		
Housewife/not working	103	27.5
Governmental employee	160	42.6
Private sector employee	85	22.7
Business/Trading	8	2.1
Students	13	3.5
Retired	6	1.6
Family income (Saudi riyals/month) (N = 362)		
<3,000	99	27.3
3,000–10,999	153	42.3
11,000–15,999	67	18.5
≥16,000	43	11.9

Table 2 shows that about two-thirds (252, 67.2%) of the participants knew that monkeypox is an infectious disease and 206 (54.9%) could recognize that it is a viral disease. Approximately 186 (49.6%) participants knew that people with monkeypox can transmit the disease to others, and 166 (44.3%) recognized that there are few cases recorded in Saudi Arabia. On the other hand, a minority of the participants knew correctly that Monkeypox cases are increasing in the United States and Europe (71, 18.9%), the chickenpox vaccine taken during childhood does not protect from monkeypox (67, 17.9%), a smallpox vaccine is available that can be used for monkeypox (64, 17.1%), a monkeypox vaccine is available in Saudi Arabia (63, 16.8%), there no specific treatment for monkeypox (56, 14.9%), and Monkeypox is a not a new infection that appeared in 2022 (53, 14.1%) (Table 2).

**Table 2:** Responses of the participants to knowledge questions and statements about monkeypox. N: Number;%: percentage

Questions	Right answers		
	Response	N	%
What kind of disease does monkeypox cause?			
Chronic disease	No	153	40.8
Immune disease	No	90	24.0
Infectious disease	Yes	252	67.2
Hereditary disease	No	215	57.3
Inflammatory disease	No	78	20.8
Metabolic disease	No	111	29.6
Monkeypox is a new infection that appeared in 2022	No	53	14.1
Monkeypox is a sexually transmitted disease	Yes	113	30.1
Monkeypox and chickenpox are the same disease	No	139	37.1
Monkeypox is common in Middle Eastern countries	No	129	34.4
Monkeypox is common in West and Central African countries	Yes	151	40.3
There are many cases recorded in Saudi Arabia	No	166	44.3
Monkeypox cases are increasing in the United States and Europe	Yes	71	18.9
Monkeypox is a contagious viral disease	Yes	206	54.9
Monkeypox is a contagious bacterial disease	No	100	26.7
Monkeypox is easily transmitted from one person to another	Yes	124	33.1
Monkeypox is transmitted to humans through the bites and scratches from infected animals	Yes	129	34.4
People with monkeypox can transmit the disease to others (the disease is transmitted between humans)	Yes	186	49.6
Monkeypox is spread by droplets (coughing and sneezing)	Yes	107	28.5
The first symptoms of monkeypox are similar to the flu	Yes	115	30.7
Skin rash is a symptom of monkeypox	Yes	178	47.5
Monkeypox only affects males	No	165	44.0
Hand sanitizers and face masks are important in preventing monkeypox	Yes	179	47.7
There is a special treatment for monkeypox	No	56	14.9
Monkeypox is spread through bodily fluids	Yes	92	24.5
There is a monkeypox vaccine available in Saudi Arabia	Yes	63	16.8
The chickenpox vaccine I got in childhood protects me from monkeypox	No	67	17.9
There is a smallpox vaccine that can be used for monkeypox	Yes	64	17.1

**Figure 1:**
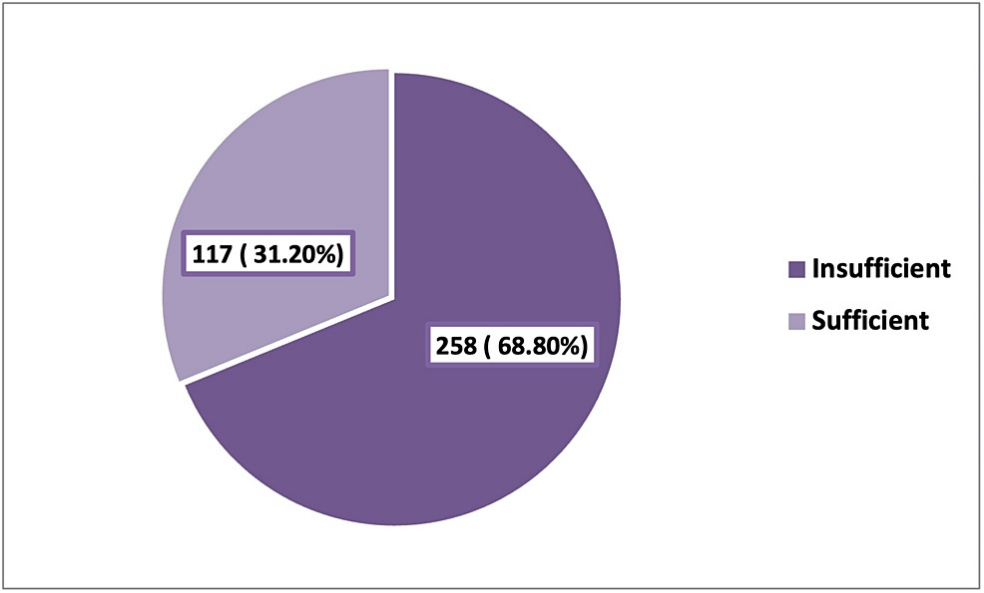
Level of knowledge of the adult general population who attended the outpatient clinics in Riyadh (Saudi Arabia) about monkeypox.

Overall, 258 (68.8%) participants had insufficient knowledge about monkeypox, as illustrated in Figure 1.

Participants aged over 45 years had a higher rate of insufficient knowledge about monkeypox compared to those aged between 18 and 25 years (43 versus 32, 81.1% versus 62.5%) (p = 0.030). University-educated/postgraduate participants had a higher rate of sufficient knowledge about monkeypox compared to those with an educational level below high school (88 versus 5, 36.8% versus 15.6%) (p = 0.006). Regarding participants’ jobs, the highest rate of sufficient knowledge was observed among students (7, 53.8%), whereas the lowest rate was observed among private sector employees (19, 22.4%) (p = 0.014). Family income of the participants was significantly associated with their level of knowledge about monkeypox as 31 (46.3%) of those whose income ranged between 11,000 and 15,999 Saudi riyals/month compared to 39 (25.5%) participants whose income ranged between 3,000 and 10,999 Saudi riyals/month had sufficient knowledge about monkeypox (p = 0.008) (Table 3).

**Table 3:** Factors associated with knowledge of the participants about monkeypox: bivariate analysis. *: chi-square test; **: Fisher’s exact test; p-values <0.05 are considered significant.

Variables	Level of knowledge about monkeypox		P-value
	Sufficient, N = 117, N (%)	Insufficient, N = 258, N (%)	
Age in years			0.030*
18–25 (N = 52)	20 (38.5)	32 (61.5)	
26–45 (N = 270)	87 (32.2)	183 (67.8)	
>45 (N = 53)	10 (18.9)	43 (81.1)	
Sex			0.630*
Male (N = 195)	63 (32.3)	132 (67.7)	
Female (N = 180)	54 (30.0)	126 (70.0)	
Place of residence			0.369**
Urban area (N = 371)	115 (31.0)	256 (69.0)	
Rural area (N = 4)	2 (50.0)	2 (50.0)	
Nationality			0.697*
Saudi (N = 349)	108 (30.9)	241 (69.1)	
Non-Saudi (N = 26)	9 (34.6)	17 (64.5)	
Marital status			0.331*
Single (N = 118)	43 (36.4)	75 (63.6)	
Married (N = 230)	66 (28.7)	164 (71.3)	
Divorced/Widowed (N = 27)	8 (29.6)	19 (70.4)	
Level of education			0.006*
Below high school (N = 32)	5 (15.6)	27 (84.4)	
High school/Intermediate diploma (N = 104)	24 (23.1)	80 (76.9)	
University/Higher education (N = 239)	88 (36.8)	151 (63.2)	
Job			0.014*
Housewife/not working (N = 103)	24 (23.3)	79 (76.7)	
Governmental employee (N = 160)	63 (39.4)	97 (60.6)	
Private sector employee (N = 85)	19 (22.4)	66 (77.6)	
Business/trading (N = 8)	2 (25.0)	6 (75.0)	
Students (N = 13)	7 (53.8)	6 (46.2)	
Retired (N = 6)	2 (33.3)	4 (66.7)	
Family income (Saudi riyals/month) (N = 362)	N = 113	N = 249	0.008*
<3,000 (N = 99)	26 (26.3)	73 (73.7)	
3,000–10,999 (N = 153)	39 (25.5)	114 (74.5)	
11,000–15,999 (N = 67)	31 (46.3)	36 (53.7)	
≥16,000 (N = 43)	17 (39.5)	26 (60.5)	

Multivariate logistic regression analysis summarized the determinants of insufficient knowledge about monkeypox after controlling for confounders. Considering participants in the age group of 18-25 years as a reference category, those aged over 45 years were at an almost three-fold risk of having insufficient knowledge about the disease (adjusted odds ratio (AOR) = 3.03, 95% confidence interval (CI) = 1.07-8.69, p = 0.037). University/higher educated participants were at 61% lower risk of having an insufficient level of knowledge about the disease compared to those with an education level of below secondary school (AOR = 0.39, 95% CI = 0.09-0.65, p = 0.013). Compared to housewives/non-working participants, governmental employees were at a significantly lower risk of having insufficient knowledge about monkeypox (AOR = 0.48, 95% CI = 0.26-0.89, p = 0.020). Family income was not significantly associated with the level of knowledge about monkeypox (Table 4).

**Table 4:** Determinants of insufficient knowledge about monkeypox among the participants: multivariate logistic regression analysis. ^a^: Reference category; p-values <0.05 are considered significant. Family income was removed from the final regression model (not significant).

Variables	Adjusted odds ratio	95% confidence interval	P-value
Age in years			
18–25^a^	1.0	---	
26–45	1.57	0.73-3.41	0.251
>45	3.05	1.07-8.69	0.037
Level of education			
Below high school^a^	1.0	---	
High school/intermediate	0.72	0.24-2.14	0.555
Diploma			
University/higher education	0.39	0.09-0.65	0.013
Job			
Housewife/not working^a^	1.0	---	
Governmental employee	0.48	0.26-0.89	0.020
Private sector employee	1.24	0.60-2.54	0.560
Business/trading	0.72	0.13-4.10	0.712
Students	0.45	0.12-1.69	0.238
Retired	0.29	0.04-1.86	0.190

**Figure 2:**
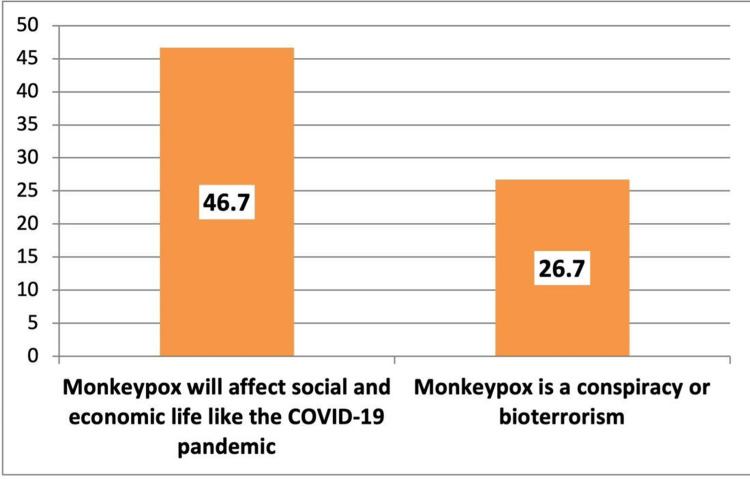
Beliefs of the participants regarding monkeypox (percentage).

Figure 2 shows that 46.7% of the participants thought that monkeypox would affect social and economic life as the COVID-19 pandemic while only 26.7% believed that monkeypox is a conspiracy or bioterrorism (Figure 2).

## Discussion

According to the World Health Organization, insufficient knowledge about monkeypox is a challenge met by authorities in conducting effective preventive programs [18]. Furthermore, exploring the knowledge of the general population about monkeypox is rarely done worldwide, with Saudi Arabia being no exception as relatively few studies have been conducted [13].

In this study, most of the surveyed participants (258, 68.8%) expressed insufficient knowledge about monkeypox. Other studies have also reported insufficient knowledge about monkeypox among the general population. In a recent population-based Saudi study, 52% of the participants expressed inadequate knowledge [13]. In the United Arab Emirates (2022), only 22.8% of university students had good knowledge about monkeypox [9]. In Pakistan (2022), most university students (68.3%) did not know about monkeypox before 2022, and overall, 76.7% of them had average knowledge [19]. In Nigeria (2022), the majority of the general adult population (89%) was aware of monkeypox while only 58.7% had good knowledge about the disease. The knowledge gaps were regarding the incubation period of the disease, main signs and symptoms, mode of transmission, and preventive measures [20]. In Bangladesh (2022), 66.6% of the general population had insufficient knowledge about many aspects of monkeypox, including the mode of transmission and signs and symptoms. Furthermore, the majority were not knowledgeable about the treatment [21]. In Indonesia (2020), only 36.5% of the general population had good knowledge about monkeypox [22]. Most medical students (72%) in another Saudi study had inadequate knowledge about monkeypox [23]. Furthermore, a considerable proportion of physicians in Saudi Arabia had poor knowledge regarding the endemic nature, mode of transmission, clinical variation from similar viral diseases, therapeutic management, and vaccination for monkeypox [24]. This can be partially explained by utilizing a different more scientific tool to assess the knowledge of physicians and medical students.

In this study, insufficient knowledge about monkeypox was higher among older participants (aged over 45 years) while higher-educated participants were less likely to have insufficient knowledge about the disease. Additionally, governmental employees were less likely to have insufficient knowledge compared to housewives/non-working participants. In accordance with our study, a similar study from Saudi Arabia showed that age, educational level, job status, and being a healthcare worker were among the determinants of the level of knowledge of the general population about monkeypox [13]. Among University students in the United Arab Emirates, being older, females, having a history of human chickenpox infection, and receiving information on human monkeypox disease during the study period were significant predictors of knowledge about monkeypox [9]. Among University students in Pakistan, the academic degree, type of study, and region of respondents were significantly associated with the level of knowledge about monkeypox [19]. Among the general population in Nigeria, males and highly educated people were more likely than their peers to express good knowledge about monkeypox [20], while in Bangladesh, education level and job status were significantly associated with the level of knowledge about monkeypox [21]. Utilizing different instruments to assess knowledge about monkeypox as well as participants’ demographic characteristics can partially explain the variation between the findings of these studies. However, common points were observed such as higher education and working status.

There are some limitations of this study. The study was conducted among people attending one healthcare facility in Riyadh (First Cluster PHCCs), which could impact the ability to generalize its findings to other facilities. Moreover, the cross-sectional design is considered another limitation as the dependent and independent variables were investigated at the same time. Despite these limitations, the study is among the few to investigate knowledge of the general population about this important health threat in Saudi Arabia which could have public health importance from a prevention point of view.

## Conclusions

Generally, knowledge of the general population about monkeypox in Riyadh, Saudi Arabia is insufficient, particularly regarding the facts that a smallpox vaccine is available that can be used for monkeypox disease, a monkeypox vaccine is available in Saudi Arabia, no specific treatment is available for monkeypox, and it is not a new disease that appeared in 2022. Older people, those with low education levels, and those not working/housewives were more likely to have insufficient knowledge about the disease. Almost half of the participants thought that monkeypox would affect social and economic life as the COVID-19 pandemic, and about a fourth believed that the disease is a conspiracy or bioterrorism. Therefore, there is a need to implement educational sessions at primary care centers and outpatient clinics of hospitals about the disease. Further nationwide studies are needed to explore the situation throughout the country.
